# Electroacupuncture Reduces Cocaine-Induced Seizures and Mortality in Mice

**DOI:** 10.1155/2013/134610

**Published:** 2013-04-17

**Authors:** Yi-Hung Chen, Boris Ivanic, Chieh-Min Chuang, Dah-Yuu Lu, Jaung-Geng Lin

**Affiliations:** ^1^Graduate Institute of Acupuncture Science, China Medical University, Taichung 40402, Taiwan; ^2^Children's University Hospital, Limbova 1, Bratislava, Slovakia; ^3^Graduate Institute of Neural and Cognitive Sciences, China Medical University, Taichung 40402, Taiwan; ^4^School of Chinese Medicine, China Medical University, Taichung 40402, Taiwan

## Abstract

The aims of this study were to characterize the protective profile of electroacupuncture (EA) on cocaine-induced seizures and mortality in mice. Mice were treated with EA (2 Hz, 50 Hz, and 100 Hz), or they underwent needle insertion without anesthesia at the Dazhui (GV14) and Baihui (GV20) acupoints before cocaine administration. EA at 50 Hz applied to GV14 and GV20 significantly reduced the seizure severity induced by a single dose of cocaine (75 mg/kg; i.p.). Furthermore, needle insertion into GV14 and GV20 and EA at 2 Hz and 50 Hz at both acupoints significantly reduced the mortality rate induced by a single lethal dose of cocaine (125 mg/kg; i.p.). In the sham control group, EA at 50 Hz applied to bilateral Tianzong (SI11) acupoints had no protective effects against cocaine. In addition, EA at 50 Hz applied to GV14 and GV20 failed to reduce the incidence of seizures and mortality induced by the local anesthetic procaine. In an immunohistochemistry study, EA (50 Hz) pretreatment at GV14 and GV20 decreased cocaine (75 mg/kg; i.p.)-induced c-Fos expression in the paraventricular thalamus. While the dopamine D_3_ receptor antagonist, SB-277011-A (30 mg/kg; s.c), did not by itself affect cocaine-induced seizure severity, it prevented the effects of EA on cocaine-induced seizures. These results suggest that EA alleviates cocaine-induced seizures and mortality and that the dopamine D_3_ receptor is involved, at least in part, in the anticonvulsant effects of EA in mice.

## 1. Introduction

### 1.1. Cocaine-Induced Seizures and Death

 Cocaine is a widely abused psychomotor stimulant [1, 2]. In addition to its addiction liability, the abuse of cocaine is associated with an array of medical complications [3]. Generalized tonic-clonic seizures and status epilepticus are well-documented neurologic sequelae of cocaine abuse [3, 4]. Seizures may be induced by cocaine after an accidental overdose or after recreational use of relatively low doses of cocaine [5, 6]. The incidence of seizures in cocaine users has been reported to be 2–10% [7]. High doses of cocaine have been associated with a disturbingly high number of sudden deaths in adults; a recent study documented that 3.1% of all sudden deaths in Southwest Spain were related to the use of cocaine [8]. Seizures are considered to be a major determinant of cocaine-related lethality in humans [4] and animals [9]. Sudden death in cocaine abuse may also be attributed to cardiac arrhythmia and intracerebral hemorrhage [10, 11]. Currently, no specific treatment modalities offer protection against cocaine-induced seizures and death in humans.

### 1.2. Acupuncture

Acupuncture has been used in traditional Chinese medicine for over 2,500 years [12]. The practice has recently gained popularity and is becoming an alternative and/or complementary treatment modality for a variety of disorders worldwide [13]. In 1997, the US National Institutes of Health stated that acupuncture is a useful method for treating many conditions and has fewer side effects as compared with other contemporary medical treatments, such as surgery or drugs [13]. Two different strategies are used in acupuncture therapy: manual acupuncture (MA) and electroacupuncture (EA). EA is a modified form of traditional MA. The advantage of EA is in its combined therapeutic effects of transcutaneous electric nerve stimulation (TENS) and MA. Most studies use EA because the latter can be standardized by frequency, voltage, waveform, length, and so forth [14]. Studies on animals and humans have demonstrated that acupuncture results in multiple biological responses [15, 16]. The best characterized mechanism is via the endogenous opioid peptides and their receptors [17].

### 1.3. Treating Epilepsy with Acupuncture

Acupuncture has been used to treat epilepsy in China for thousands of years. Many reports have suggested that acupuncture, TENS and other alternative therapies may produce positive effects in epilepsy [19], although some studies disagree [20]. The major advantage of acupuncture is the absence of side effects. In the clinic, acupuncture at specific acupoints such as the Zusanli (ST36), Dazhui (GV14), and Baihui (GV20) has significantly ameliorated symptoms of epilepsy [21–23]. Although some evidence supports this contention [24–26], the precise mechanism remains unknown. It is noted that acupuncture to GV20 point has been used to treat loss of consciousness and tinnitus, in addition to relief of mental abstraction, sluggish speech, and hysteria [27]. Recent studies have shown that EA inhibits seizures in experimental rat models. These protective effects may be related to increased concentrations of inhibitory amino acids [28], decreased levels of nitric oxide in the central nervous system (CNS) [26, 29], or increased cellular glutamic acid decarboxylase-67 mRNA expression, thereby increasing the production of *γ*-aminobutyric acid (GABA) [30]. The possibility that EA protects against cocaine-induced seizures and death has not yet been explored.

### 1.4. Acupuncture and Abused Drugs

Acupuncture has been widely used throughout Asian countries for treating many functional disorders, such as substance abuse and mental illness [31]. Some preclinical data have shown that acupuncture can modify the morphine withdrawal syndrome and suppress alcohol drinking behavior in rats [32, 33]. Further evidence suggests that acupuncture at a specific acupoint (Shenmen; HT7) attenuates the ethanol-induced dopamine release in the nucleus accumbens through the GABA_B_ receptor [34] and suppresses c-Fos expression in the nucleus accumbens and ventromedial striatum following a nicotine challenge in rats sensitized to nicotine [35].

### 1.5. c-Fos Expression

Expression of *c-fos *gene or Fos protein is widely used as a marker to identify neuronal pathways involved in the integration of noxious inputs [36–40]. High doses of cocaine have been reported to induce *c-fos* mRNA expression in many brain regions [41].

In this study, EA experiments were conducted to (1) establish the applicability of EA to cocaine-induced seizure and mortality in a mouse model, (2) determine the frequency-dependent antiseizure activity of EA, and (3) correlate the efficacy of EA with the expression of Fos protein in the brain.

## 2. Methods

### 2.1. Laboratory Animals

Male ICR mice (28–35 g; BioLasco Taiwan Co., Ltd., Taiwan) were housed under a 12:12 h light/dark cycle with food and water available *ad libitum* in our animal facility for at least 4 days prior to the experiments, which were conducted between 10:00 and 17:00 h. The experimental procedures were approved by the China Medical University Institutional Animal Care and Use Committee, in accordance with the Chinese Taipei Society of Laboratory Animal Sciences guidance on care and use of laboratory animals. Experiments were designed to keep the number of mice at a minimum and care was taken to minimize suffering.

### 2.2. Electroacupuncture

For the EA treatment, animals were covered by paraffin film and restrained by tapes without anesthesia. A pair of stainless steel acupuncture needles (Tianjin HaingLimSou Won Medical CO., Ltd.; Gauge 40) were inserted 3-4 mm deep into the murine equivalent of the human GV14 and GV20, that is, (1) the skin at the location between the last cervical and the first thoracic vertebral spinous processes at the midline of the back, which is equivalent to the human GV14 and (2) at the vertex of the parietal bone, that is, the midpoint of the connecting line between the auricular apices, which is equivalent to the human GV20 acupoints [42, 43]. EA stimuli were delivered by an EA Trio 300 stimulator (Ito, Japan) at 1 mA intensities for a 15 min duration at a frequency of 2, 50, or 100 Hz, with a pulse width of 150 *μ*s. The two electrodes were connected to the needles, which were inserted into GV20 and GV14. In the control group, animals were also covered by paraffin film and restrained by tape for 15 min [44]. A sham EA was performed by bilateral insertion of a pair of stainless steel acupuncture needles approximately 3-4 mm deep into the middle of each scapula, which is equivalent to the human Tianzong (SI11).

### 2.3. Behavioral Study

Following the injection of cocaine or saline, animals were placed in a plastic observation box and seizure scores were assessed by the Itzhak scale, which categorizes five stages according to their severity over a 30 min period [18]. The Itzhak stages of seizure are as follows.

**Stage 1**

Normal behavior (moving about the cage, sniffing, and rearing).

**Stage 2**

Hyperactivity (running movement characterized by rapid changes in position).

**Stage 3**

Animal remains in the same place for several seconds with fast repetitive movements of the head, face, mouth, or forelimb, as well as head nodding.

**Stage 4**

Forelimb clonus and rearing.

**Stage 5**

Full motor seizures, characterized by clonus of forelimbs and hindlimbs, flexion of head or entire body, and complete loss of postural control.

### 2.4. Immunohistochemistry Procedure

The immunohistochemistry procedure was similar to that described by Inan et al. [45]. Two hours after the injection of saline or cocaine, animals were deeply anesthetized with urethane (1.2 g/kg; i.p.) and perfused intracardially with ice-cold 0.1 M phosphate-buffered saline (PBS) followed by 4% paraformaldehyde in 0.1 M PBS. The brains were removed, postfixed for 2 h, and kept in 30% sucrose solution overnight. Brain sections of 50 *μ*m thickness were cut with a cryostat (LEICA CM 3050). Free-floating sections were stored in PBS at 4°C until immunohistochemistry was performed. Three brain sections, from −2.255 mm to −1.856 mm caudal to bregma, were randomly selected for immunohistochemistry procedures. Tissues were processed for c-Fos immunoreactivity by the avidin-biotin complex procedure [46]. Tissues were initially treated with 3% H_2_O_2_ to reduce endogenous peroxidase activity, then washed twice for 10 min with PBS and blocked with 20% normal goat serum (1 : 20) for 2 h at room temperature. The sections were then incubated on a shaker for 2 d at 4°C with a rabbit c-Fos antibody (1 : 1000 dilution) (sc-52; Santa Cruz, USA). After thorough rinsing, sections were incubated in biotinylated anti-rabbit immunoglobulin G secondary antibody (1 : 300 dilution; Vector Laboratories, USA) for 2 h at room temperature. Following two 10 min PBS rinses, the sections were incubated in a complex of avidin-biotin-peroxidase in 1 : 300 solution at room temperature for 90 min (Vectastain ABC Elite kit, Vector Laboratories). Following three 10 min washes in Tris-buffered saline, the sections were placed in a 0.05% diaminobenzidine (Sigma)/0.001% H_2_O_2_ solution for 4-5 min and washed again with Tris-buffered saline 3 times for 10 min each. Sections were mounted on slides with 0.25% gel alcohol, air-dried, and dehydrated with graded alcohol (50%, 70%, 95%, and 100%, for 6 min each) followed by xylene (3 times for 10 min each), and the coverslip was placed using Permount. c-Fos positive nuclei were observed under a light microscope and counted at 200x magnification. c-Fos immunoreactive nuclei were counted on the captured images using MetaMorph (Universal Imaging Corp.) software. Bilateral calculations were performed on the paraventricular thalamus, amygdala area, and caudoputamen. The number of immunoreactive nuclei was averaged for each mouse.

### 2.5. Drugs and Chemicals

Cocaine-HCl was purchased from the National Bureau of Controlled Drugs, Department of Health, Taipei, Taiwan. Freshly dissolved in 0.9% NaCl, all test agents were administered in a dose/volume of 10 mg/mL. Cocaine at the dose of 75 mg/kg causes clonic seizures in mice [48]. Cocaine at 125 mg/kg is considered to be a lethal dose [49]. In this study, cocaine at 75 mg/kg and 125 mg/kg was injected intraperitoneally to induce seizures and death. Procaine HCl was purchased from Sigma Chemical Co. (St. Louis, MO, USA). Procaine (250 mg/kg and 400 mg/kg; i.p.) was used to induce seizures and death in mice, respectively [50]. SB-277011-A (30 mg/kg; s.c.) was purchased from Tocris Bioscience (Ellisville, MO, USA) and was used to block the dopamine D_3_ receptor [51].

### 2.6. Statistical Analysis

Data were expressed as means and the standard error of the mean (SEM). Group comparisons for seizure scores and the number of cocaine-induced c-Fos positive nuclei were evaluated by one-way ANOVA, followed by Tukey's post-hoc test. Group comparisons for mortality rates and seizure incidence were evaluated by chi-square test or Fisher's exact test. Significance was considered at *P* < 0.05 for all tests. Statistical analysis was performed by SPSS version 18.0 (SPSS Inc., Chicago, IL, USA).

## 3. Results

### 3.1. Positioning of Acupuncture Needles

**Figure 1:**
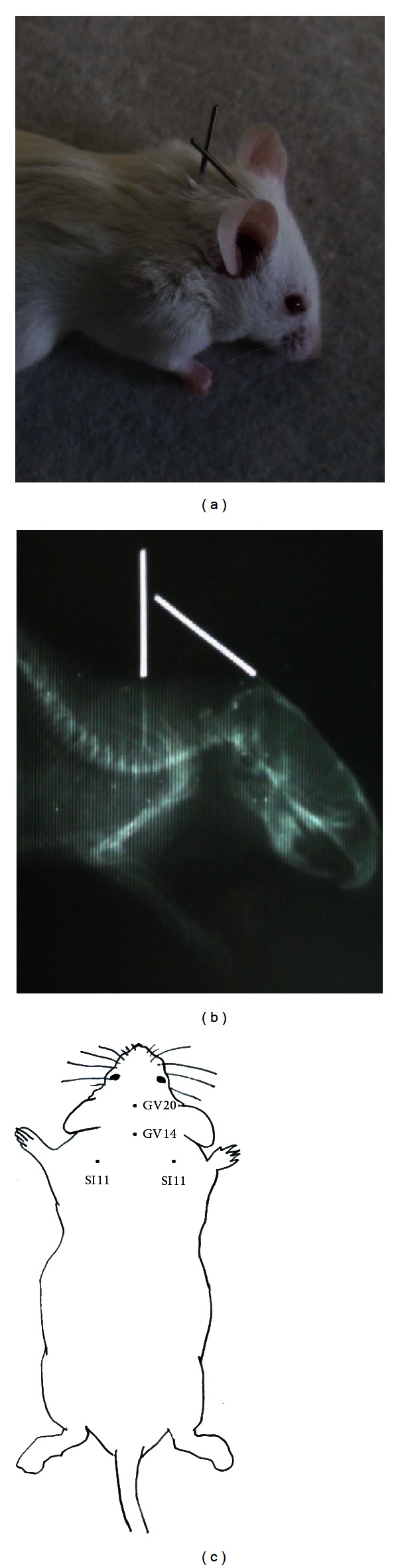
Photograph (a) and X-ray (b) of a mouse under anesthesia with acupuncture needles inserted into GV14 and GV20 acupoints. (c) Mouse schematic showing the location of the acupoints used in the study.

We used X-ray images to ensure accurate positioning of acupuncture needles. As shown in Figure 1(a), a mouse was anesthetized with urethane and two acupuncture needles were inserted into the GV14 and GV20 acupoints.  Figure 1(b) shows the position of the acupuncture needles, as revealed by X-ray.  Figure 1(c) shows the positions of GV14, GV20, and SI11 in mice. 

### 3.2. Effects of EA on Cocaine-Induced Seizures

Firstly, the effects of EA were examined on seizures induced by a single dose of cocaine (75 mg/kg) in six groups of mice (a control group, three EA treatment groups, a sham EA group, and a needle insertion group). In the control group, animals were restrained with the same procedure as that used with the EA group but without EA. In the EA treatment groups, EA (2 Hz, 50 Hz, and 100 Hz) was applied for 15 min to GV14 and GV20 acupoints prior to cocaine injection. In the sham EA group, mice received EA (50 Hz) applied to bilateral SI11. In the needle insertion group, needles were inserted into GV14 and GV20 acupoints, but without electrical stimulation. After undergoing control, EA, sham, or needle insertion treatment, all animals received an intraperitoneal injection of cocaine (75 mg/kg). Seizure severity was measured by the 5-stage cocaine seizure scale published by Itzhak [18].

**Figure 2:**
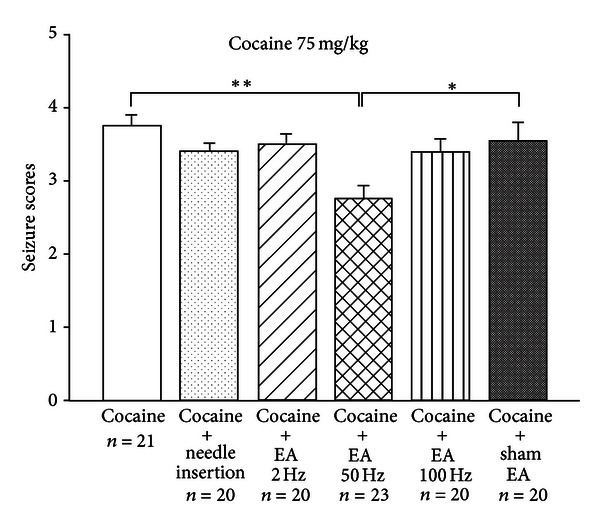
Effects of EA at different frequencies (2 Hz, 50 Hz, and 100 Hz), needle insertion exerted at GV14 and GV20 acupoints, and sham EA (50 Hz; at bilateral SI11) on cocaine-induced seizures. Mice were administered with cocaine (75 mg/kg; i.p.) at 1 min after EA or needle insertion. Seizure severity was measured by the Itzhak five-stage scale [18]. Between-group comparisons for each group were performed by one-way ANOVA followed by Tukey's test (*P < 0.05; **P < 0.01; *n*: the number of animals).

As shown in Figure 2, the average seizure score was significantly lower in animals treated with EA (50 Hz) compared with those in the control and sham EA groups. No such effects were seen with EA 2 Hz, EA 100 Hz, or needle insertion. It appears that EA 50 Hz at the GV14 and GV20 acupoints significantly reduced seizure scores induced by a single cocaine administration (75 mg/kg), whereas EA at 2 Hz and 100 Hz had no such effect. Furthermore, cocaine-induced seizures were not significantly affected by either 15 min of needle insertion into GV14 and GV20 or by EA at SI11.

Table 1 depicts the incidence of seizures (higher or equal to stage 3) induced by cocaine (75 mg/kg; i.p.). Pretreatment of animals with EA (50 Hz) significantly reduced seizure incidence.

### 3.3. Effects of EA on Cocaine-Induced Death

The effects of EA on death induced by single administrations of a high dose of cocaine (125 mg/kg) were investigated in a series of six groups, that is, control group, three EA treatment groups, a sham EA group, and a needle insertion group. After undergoing treatment, all animals received an intraperitoneal injection of cocaine (125 mg/kg).

**Figure 3:**
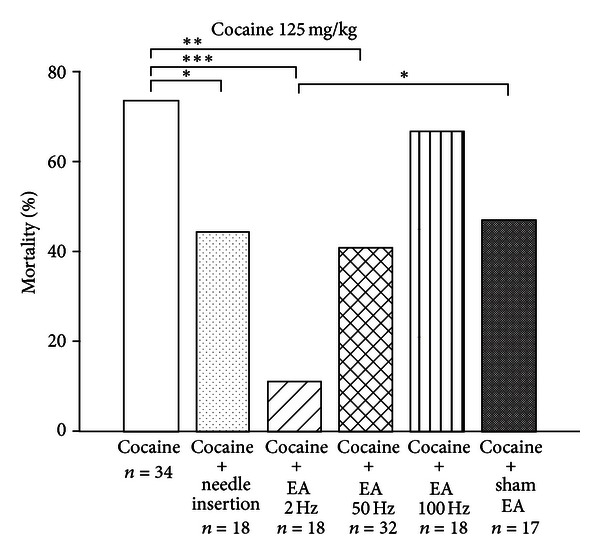
Effects of EA at different frequencies (2 Hz, 50 Hz, and 100 Hz), needle insertion exerted at GV14 and GV20 acupoints, and sham EA (at bilateral SI11; 50 Hz) on cocaine-induced mortality rates. Mice were administered with cocaine (125 mg/kg; i.p.) at 1 min after EA or needle insertion and mortality was monitored for 1 hour. Between-group comparisons for each group were performed by chi-square test (*P < 0.05; **P < 0.01;***P < 0.001; *n*: the number of animals).

As shown in Figure 3, mortality rates were significantly lower in the needle insertion and EA (2, 50 Hz) groups as compared to mortality in the control group. The EA (2 Hz) group mortality rate was also lower than that of the sham EA group. Needle insertion into GV14 and GV20, as well as EA at 2 Hz and 50 Hz applied to GV14 and GV20, significantly reduced the mortality rate induced by a single dose of cocaine (125 mg/kg). However, EA at 100 Hz had no such effect. EA at SI11 as a sham control reduced the mortality rate induced by cocaine but did not reach a statistically significant level.

### 3.4. Effects of EA on Procaine-Induced Seizure and Death

In addition to inhibiting dopamine uptake, cocaine acts as a local anesthetic by blocking voltage-dependent Na^+^ channels [52, 53]. Local anesthetics, such as procaine, can cause CNS and cardiovascular toxicity when plasma concentrations are increased by accidental intravenous injection of a lethal dose of procaine [54]. However, procaine has no CNS stimulant effects compared with those of cocaine. Therefore, the effects of EA on procaine-induced seizures and death were examined.

**Figure 4:**
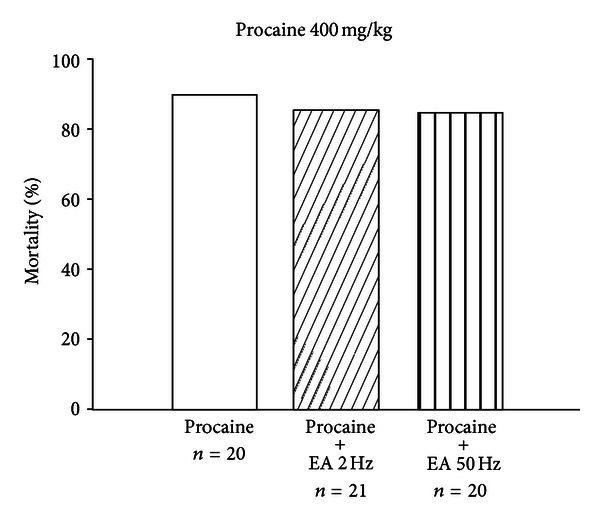
Effects of EA at 2 Hz and 50 Hz exerted at GV14 and GV20 acupoints on procaine-induced (400 mg/kg, i.p) mortality rates. At 1 min after EA, procaine was injected and the mortality was monitored for 1 hour. Between-group comparisons for each group were performed by chi-square test or Fisher's exact test. No significant between-group differences were observed (*n*: the number of animals).

The seizure incidence of procaine-treated mice (250 mg/kg; i.p.) is shown in Table 1. When animals were pretreated with EA (50 Hz) at acupoints GV14 and GV20, the incidence was not affected. Furthermore, as shown in Figure 4, mortality rates did not differ significantly between the control group and EA (2 and 50 Hz) groups. It appears that EA at 2 Hz and 50 Hz, when applied to GV14 and GV20, fails to affect the mortality rate associated with procaine (400 mg/kg; i.p.) in mice.

### 3.5. EA Attenuation of Cocaine-Induced c-Fos Expression

This series of studies was undertaken to test the effects of EA on cocaine-induced (75 mg/kg) c-Fos expression in the mouse brain. Mice were divided into 4 groups: saline control, EA alone, cocaine, and cocaine plus EA. 

All mice in the saline control group and cocaine group were restrained without EA. Mice in the EA alone and cocaine plus EA groups received EA (50 Hz) at GV14 and GV20 acupoints. Following the restraining procedure or EA, the mice were allowed to recover for 1 min, before being injected with saline (saline control and EA-alone groups) or cocaine (cocaine group and cocaine plus EA groups).

**Figure 5:**
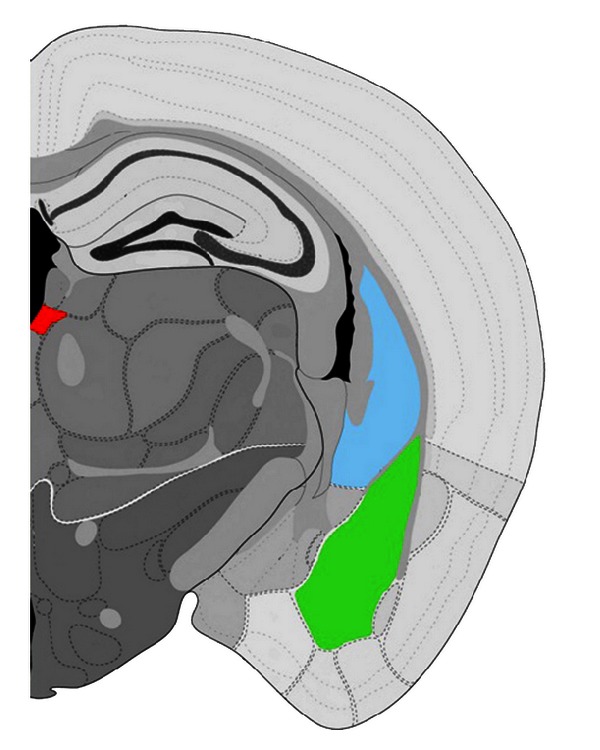
Schematic representation of the brain regions in which the c-Fos immunoreactive neurons were counted. The paraventricular thalamus (in red), the caudoputamen (blue), and the amygdala area (including lateral amygdalar nucleus, basolateral amygdalar nucleus, and basomedial amygdalar nucleus; green) are indicated. The drawing has been modified from the Allen Brain Atlas online database (http://www.brain-map.org/; [47]).

**Figure 6:**
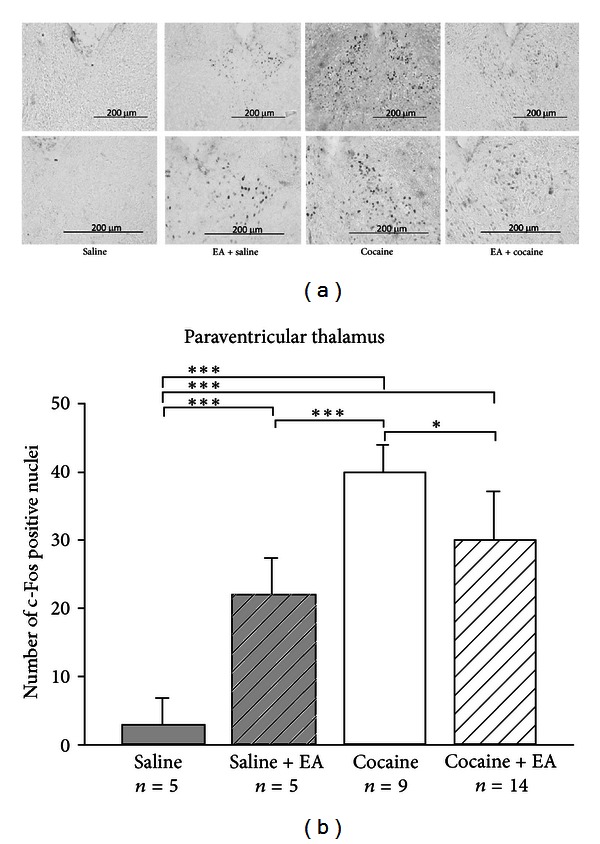
(a) Representative photomicrographs of c-Fos expression induced by cocaine in the paraventricular thalamus and the effects of EA. c-Fos expression was not observed among saline controls, whereas c-Fos expression was evident in the paraventricular thalamus among animals in the EA-alone, cocaine-alone (75 mg/kg; i.p.), and cocaine plus EA groups. (b) Effects of EA on controls and cocaine-induced c-Fos expression in the paraventricular thalamus. The numbers of c-Fos positive nuclei were counted and averaged from three randomly chosen sections from each animal in each group. EA at 50 Hz was applied to the GV14 and GV20 acupoints. EA increased c-Fos expression in the paraventricular thalamus, while EA decreased the number of c-Fos positive nuclei induced by cocaine. Between-group comparisons for each group were performed by ANOVA, followed by Tukey's test (*P < 0.05; ***P < 0.001; *n*: the number of animals).

**Figure 7:**
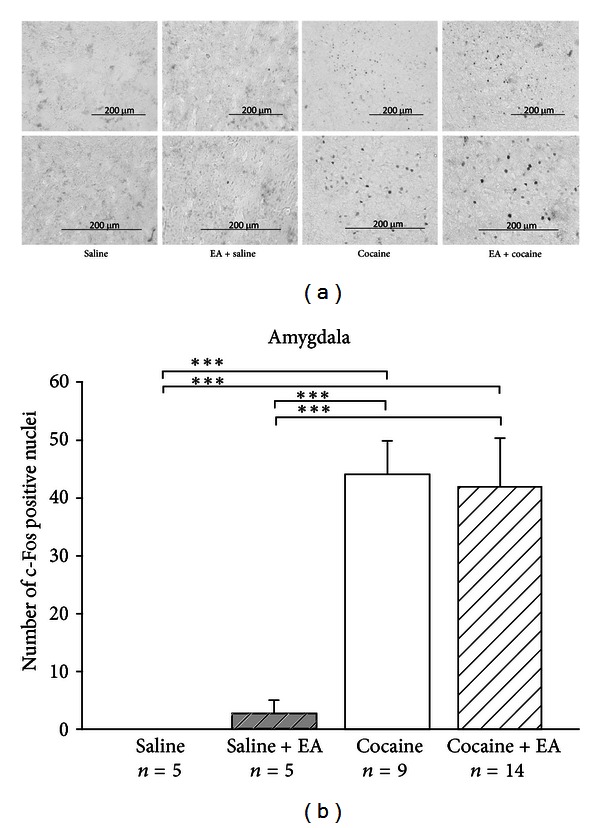
(a) Representative photomicrographs of c-Fos expression induced by cocaine in the amygdala and the effects of EA. Whereas c-Fos expression was absent among animals in the saline control and EA plus saline groups, c-Fos expression in the amygdala was observed in animals treated with cocaine (75 mg/kg; i.p.) and cocaine plus EA. (b) Effects of EA on controls and cocaine-induced c-Fos expression in the amygdala. The numbers of c-Fos positive nuclei were counted and averaged from three randomly chosen sections from each animal in each group. EA at 50 Hz was applied to the GV14 and GV20 acupoints. Cocaine administration induced a marked level of c-Fos expression, while EA did not significantly decrease the number of c-Fos positive nuclei induced by cocaine. Between-group comparisons for each group were performed by ANOVA, followed by Tukey's test (***P < 0.001; *n*: the number of animals).

**Figure 8:**
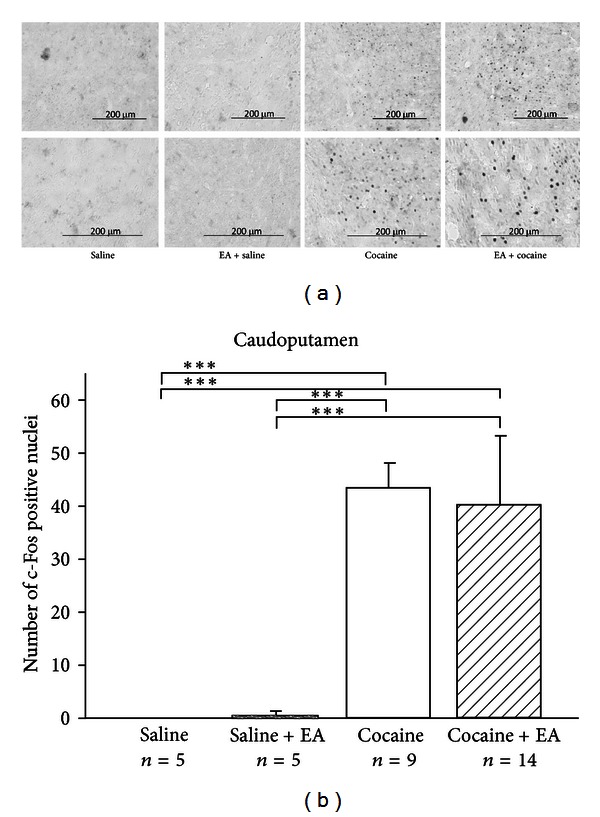
(a) Representative photomicrographs of c-Fos expression induced by cocaine in the caudoputamen and the effects of EA. Whereas c-Fos expression was not observed in the saline control and EA plus saline groups, c-Fos expression in the caudoputamen was observed among animals treated with cocaine (75 mg/kg; i.p.), and cocaine plus EA. (b) Effects of EA on controls and cocaine-induced c-Fos expression in the caudoputamen. The numbers of c-Fos positive nuclei were counted and averaged from three randomly chosen sections from each animal in each group. EA at 50 Hz was applied to the GV14 and GV20 acupoints. Cocaine administration induced a marked level of c-Fos expression, while EA did not significantly decrease the number of c-Fos positive nuclei induced by cocaine. Between-group comparisons for each group were performed by ANOVA, followed by Tukey's test (***P < 0.001; *n*: the number of animals).

Figure 5 shows the brain regions in which the c-Fos immunoreactive neurons were counted. Results are shown in Figures 6, 7, and 8. c-Fos expression was examined in the three brain areas, including paraventricular thalamus (Figure 6), amygdala area (Figure 7), and caudoputamen (Figure 8). Few c-Fos positive nuclei were found in these areas in the saline control group. In the EA-alone group, c-Fos positive nuclei were found only in the paraventricular thalamus (Figure 6). It appears that EA 50 Hz alone had no significant effect on c-Fos expression, except in the paraventricular thalamus. In the cocaine group, c-Fos positive nuclei were found in all three areas and at significantly higher levels than those seen in the saline control group. In the cocaine plus EA group, although c-Fos positive nuclei were noted in all three brain areas, c-Fos-positive nuclei were significantly decreased in the paraventricular thalamus (Figure 6) as compared to those in the cocaine group. It appears that pretreatment with EA 50 Hz significantly reduced the number of c-Fos positive cells induced by cocaine in the paraventricular thalamus, but not in the amygdala area (Figure 7) and caudoputamen (Figure 8).

### 3.6. EA Effects on Cocaine-Induced Seizures Blocked by the Dopamine D_3_ Receptor Antagonist SB-277011-A

Cocaine enhances monoamine system activity through the blockade of dopamine and serotonin reuptake. However, the mechanism of seizures induced by cocaine is complex and involves interaction of the drug with several neurotransmitter systems as well as with voltage-dependent sodium channels [55]. It is believed that dopamine receptors play an important role in cocaine-induced seizures and death. It is known that the paraventricular thalamus expresses dopamine D_3_ mRNA [56] and we found that EA induced c-Fos expression in the paraventricular thalamus. We therefore tested the possibility that dopamine D_3_ receptors may mediate the effects of EA on cocaine-induced seizures.

Four groups were included: a control group, an SB-277011-A group, an EA group, and an SB-277011-A plus EA group. In the SB-277011-A group and EA group plus SB-277011-A, the compound SB-277011-A (30 mg/kg) was administered subcutaneously for 30 min prior to the restraining procedure or EA treatment. After the restraining procedure or EA treatment, all animals received an intraperitoneal injection of cocaine (75 mg/kg). Seizure severity was measured by the Itzhak five-stage cocaine seizure scale [18].

**Figure 9:**
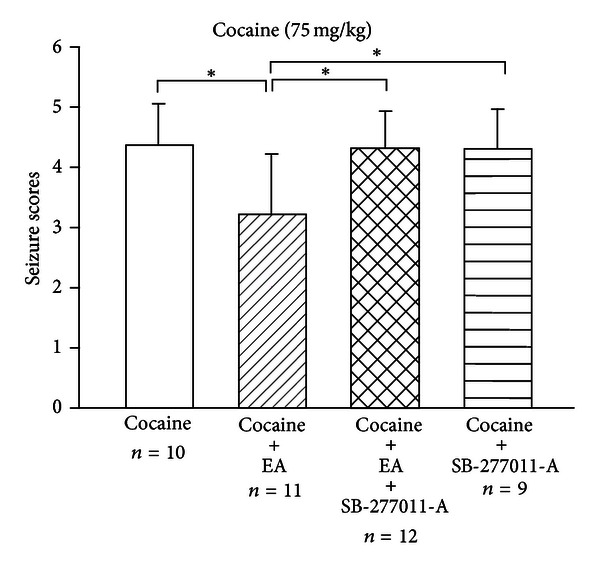
Effects of the dopamine D_3_ receptor antagonist, SB-277011-A, on the anticonvulsant effects of EA. SB-277011-A (30 mg/kg) was administered subcutaneously 30 min prior to the control restraining procedure or EA treatment. EA (50 Hz) was applied for 15 min to the GV14 and GV20 acupoints prior to cocaine injection. After the restraining procedure or EA treatment, all animals received an intraperitoneal injection of cocaine (75 mg/kg). Seizure severity was measured by the Itzhak five-stage scale [18]. Between-group comparisons for each group were performed by one-way ANOVA, followed by Tukey's test (*P < 0.05; *n*: the number of animals).

As shown in Figure 9, the EA (50 Hz) group had significantly lower average seizure scores compared with those of the remaining three groups. Notably, seizure scores in the SB-277011-A group and SB-277011-A plus EA group did not differ significantly from the control group but were significantly higher than those in the EA group. It appears that while the dopamine D_3_ receptor antagonist, SB-277011-A (30 mg/kg; s.c.), did not affect cocaine-induced seizure severity, it did prevent the effects of EA on cocaine-induced seizures. 

## 4. Discussion

### 4.1. EA Decreases Cocaine-Induced Effects

The psychostimulant and euphoric effects of cocaine are considered to be associated with the blockade of dopamine uptake in the CNS. Acupuncture has been widely used throughout Asian countries for treating various functional disorders, including substance abuse [31]. Immunohistochemical investigations by Lee et al. (2009) [57] reported that acupuncture can reduce repeated cocaine-induced locomotor activity in rats and the expression of tyrosine hydroxylase (TH) in the rat brain, which suggests that acupuncture may effectively inhibit the behavioral effects of cocaine by modulating the central dopaminergic system.

As mentioned earlier, cocaine abuse is associated with a risk of various medical complications, including seizures and death [3]. It has previously been reported that cocaine at doses ranging from 2.5 mg/kg to 20 mg/kg (i.p.) causes conditioned place preference, representing the rewarding effects of cocaine [58]. At a dose as high as 40 mg/kg (i.p.), cocaine caused seizures in mice [59]. The calculated ED_50_ value for cocaine-induced seizures is 58.84 mg/kg [59]. Cocaine at 75 mg/kg (i.p.) produces seizures in more than 90% of mice [48]. Cocaine at 125 mg/kg is considered to be a lethal dose [49]. In the present study, we found that EA at 50 Hz exerted at GV14 and GV20 acupoints significantly reduced the seizure severity induced by a single cocaine (75 mg/kg; i.p.) administration. Moreover, needle insertion into GV14 and GV20 as well as EA at 2 Hz and 50 Hz exerted at GV14 and GV20 acupoints significantly reduced the mortality rate induced by a single cocaine (125 mg/kg) administration. Conversely, EA at 50 Hz applied to bilateral SI11 acupoints had no such effects. In addition, EA failed to protect against procaine-induced seizure incidence and lethality in mice. We report here for the first time that EA reduced seizures and mortality induced by a high dose of cocaine.

### 4.2. Cocaine-Induced c-Fos Expression and Effects of EA

Expression of the *c-fos *gene or Fos protein is commonly used as a marker for neuronal activation, seizure pathways, and sites of action of neuroactive drugs [60–64]. It has been reported that an acute injection of cocaine (65 mg/kg) in rats increased expression of *c-fos* mRNA in the dentate gyrus of the hippocampus and olfactory bulb and limbic cortical regions as well as the striatum and ventromedial hypothalamic nucleus [41].

In this study, we focused on three brain areas: the paraventricular thalamus, amygdala, and caudoputamen. We found that cocaine at a dose of 75 mg/kg (i.p.) induced marked c-Fos expression in all areas. Pretreatment with 50 Hz EA significantly reduced the number of c-Fos positive cells induced by cocaine in the paraventricular thalamus, but not in the amygdala or caudoputamen.

The thalamus is considered to be an important interface between the ventral pallidum and the dorsal medial prefrontal cortex which may therefore contribute to the development of compulsive drug-seeking behavior [65]. One thalamic nucleus that is of particular interest is the paraventricular thalamus, a component of the dorsal midline thalamic group [66]. The thalamic paraventricular nucleus projects to the nucleus accumbens and other limbic sites, including the prefrontal cortex, amygdala, and hippocampus; these projections are predominantly glutamatergic [67]. The paraventricular thalamus also receives a dopaminergic innervation, in part derived from the ventral tegmental area, and paraventricular thalamus neurons expressing dopamine D_3_ mRNA [56]. The paraventricular thalamus has been implicated in stress reactivity [68], reward-seeking behavior [69], and general arousal activity [70]. Furthermore, inactivation of the paraventricular thalamus prevents context-induced reinstatement of alcohol-seeking [71] and cocaine-primed reinstatement in rats [72]. Paraventricular thalamus lesions block cocaine sensitization [67].

The paraventricular thalamus is related to seizures activity. Mraovitch and Calando [73] used immunocytochemistry to determine the regional and temporal distribution of Fos protein expression in awake and unrestrained rats after a unilateral stereotaxic microinjection of the cholinergic agonist carbachol in the thalamic ventroposterolateral and reticular nuclei, previously shown to cause limbic and generalized seizures [73]. They found that paraventricular thalamus activation occurred after 15 min after administration of epileptic agents.

The amygdala is involved in both temporal lobe epilepsy and in cocaine mechanisms in the brain. In particular, the central nucleus of the amygdala is a highly epileptogenic brain area and, of the amygdaloid nuclei, responds most rapidly to a kindling stimulus [74]. The caudoputamen is a part of the striatum area and plays an important role in control of movement in animals [75–77]. 

As shown by our results and the published literature [41], acute cocaine administration increases c-Fos expression in many brain areas. Among these areas, the paraventricular thalamus and amygdala are structures associated with seizures, while the caudoputamen is associated with movement control. We therefore analyzed c-Fos expression in these three specific regions. Our results implicate involvement of the paraventricular thalamus in the effects of EA on cocaine-induced seizures.

### 4.3. EA Induces c-Fos Expression in the Paraventricular Thalamus and Dopamine D_3_ Receptors Are Involved in the Effects of EA

Medeiros et al. (2003) reported higher levels of c-Fos expression induced by EA at the ST36 point in animals, that is, in the dorsal raphe nucleus, locus coeruleus, posterior hypothalamus, and central medial nucleus of the thalamus [78]. In the present study, we found that EA alone at GV14 and GV20 acupoints induced significant c-Fos expression in the paraventricular thalamus. 

Recent research indicates that dopamine D_3_ receptors may play an important role in cocaine-induced seizures [51]. The D_3_ antagonist SB-277011-A has been used to block dopamine D_3_ receptors, at doses ranging from 3 mg/kg to 30 mg/kg (s.c.) [18, 79]. SB-277011-A prevented the anticonvulsant effects of the D_3_/D_2_ receptor agonist (+)-PD-128,907 on cocaine-induced seizures. Notably, the protection afforded by (+)-PD-128,907 was eliminated in D_3_ receptor-deficient mice, whereas the anticonvulsant effects of (+)-PD-128,907 were preserved in D_2_ receptor knockout mice.

As mentioned above, the paraventricular thalamus expresses dopamine D_3_ mRNA, while EA alone induces c-Fos expression in the paraventricular thalamus. We therefore sought to determine whether dopamine D_3_ receptors mediate the effects of EA on cocaine-induced seizures. Our results revealed that while the D_3_ receptor antagonist SB-277011-A did not affect cocaine-induced seizure severity, it prevented the effects of EA on cocaine-induced seizures. This finding suggests that the D_3_ receptor is involved, at least in part, in the anticonvulsant effects of EA.

Interesting topics that remain for future studies include an investigation into whether intraparaventricular thalamus injection of a D_3_ receptor antagonist prevents EA-induced anticonvulsive effects and whether a D_3_ receptor antagonist antagonizes EA-induced changes to c-Fos expression in the paraventricular thalamus or has any effect on cocaine-induced mortality.

### 4.4. A Complex Mechanism Underlies Cocaine-Induced Death

It is noted that EA at 50 Hz only, when exerted at GV14 and GV20 acupoints, significantly reduced seizure severity induced by a single cocaine (75 mg/kg; i.p.) administration. However, needle insertion into GV14 and GV20 acupoints as well as EA at 2 Hz and 50 Hz effectively reduced the mortality rate induced by a single cocaine (125 mg/kg) administration. As mentioned earlier, seizures are considered to be a major determinant of cocaine-related lethality in both humans [4] and animals [9]. However, the mechanism of sudden death in cocaine abuse also includes cardiac arrhythmia and intracerebral hemorrhage [10, 11]. Cocaine produces euphoria, elation, mood elevation, alertness, attention focusing, and fatigue reduction through interactions with monoamine transporters [80]. In addition to the central effects, cocaine also blocks norepinephrine uptake and increases sympathetic activity in the periphery [81]. Overstimulation of sympathetic activity may cause cardiac arrhythmia and intracerebral hemorrhage and hence may contribute to cocaine-induced fatality [10, 11]. EA frequency-specific differences observed in protection against seizures and death may be because the mechanism that induces death is more complicated than that involved in seizures. It is recognized that acupuncture can account for different effects in the autonomic nervous system [82, 83]. For example, the GV14 acupoint is capable of increasing parasympathetic activities while simultaneously suppressing sympathetic activities [82]. This may explain why EA at 2 Hz is particularly effective against cocaine-induced death.

### 4.5. EA Frequency

The best-known mechanism of EA is via the endogenous opiates and their receptors. Different kinds of endogenous opioid peptides, such as *β*-endorphin, enkephalin, endomorphin, and dynorphin, reportedly act in a frequency-dependent manner in EA. At a low frequency (2 Hz), EA accelerated the release of *β*-endorphin and enkephalin in the CNS, whereas high-frequency EA (100 Hz) accelerated the release of dynorphin [17, 84–86]. In the present study, we found that EA applied to GV14 and GV20 acupoints at the frequency of 50 Hz is most effective in reducing seizure severity induced by a single cocaine administration. There were few reports in the literature as to the effects of EA 50 Hz as compared to those of EA 2 and 100 Hz. However, it has been recently reported that EA 60 Hz increases the pain threshold by a greater extent than any other frequency [87]. EA at a frequency of 60 Hz induced the simultaneous release of met-enkephalin, *β*-endorphin, and dynorphin-A in extensive analgesia-related nuclei and areas of the CNS, such as the periaqueductal gray, the paraventricular nucleus of the hypothalamus, the ventromedial nucleus of the hypothalamus, the dorsal raphe nucleus, and the nucleus raphe magnus, amongst others. These results suggest that EA at 60 Hz may contribute to optimal analgesic effects. The effects of EA at 50 or 60 Hz deserve to be studied further [87].

No effective treatment currently exists for cocaine-induced seizures and death. Our results suggest that EA reduces seizure severity and death caused by cocaine in an animal model of cocaine abuse. We found evidence for involvement of the dopamine D_3_ receptor, at least in part, in the anticonvulsant effects of EA.

## Figures and Tables

**Table 1:** EA (50 Hz) applied to GV14 and GV20 reduced the incidence of cocaine-induced seizures but did not alter the incidence of procaine-induced seizures.

	Control	EA 50 Hz
Cocaine (75 mg/kg; i.p.)	20/21	14/21*
Procaine (250 mg/kg; i.p.)	8/9	8/9

The proportion of mice exhibiting seizures is shown here. The effects of cocaine, alone and in combination with EA, were compared by Fisher's exact test (*P < 0.05). Similarly, the effects of procaine, alone and in combination with EA, were compared.
